# Machine Learning-Based Prediction of Functional Deficit from Anthropometric and Cardiometabolic Biomarkers in Middle-Aged and Older People

**DOI:** 10.3390/jcm15218618

**Published:** 2026-11-05

**Authors:** Jessica Mondaca Urrutia, Rodrigo Yáñez-Sepúlveda, Paz Pezoa-Fuentes, Yeny Concha-Cisternas, Eduardo Guzmán-Muñoz, Carlos Abraham Herrera-Amante, Lawrence W. Judge, Claudio Farías-Valenzuela, Pablo Valdés-Badilla, Bastian Parada-Flores, Jordan Hernandez-Martínez, Exal Garcia-Carrillo

**Affiliations:** 1 Department of Physical Activity Sciences, Faculty of Education Sciences, Universidad Católica del Maule, Talca 3480112, Chile; jmondaca@ucm.cl (J.M.U.); paz.pezoa.fu@gmail.com (P.P.-F.); valdesbadilla@gmail.com (P.V.-B.); paradafloresbastian@gmail.com (B.P.-F.); 2 Faculty of Education and Humanities, School of Sport Sciences, Universidad Andres Bello, Viña del Mar 2520000, Chile; rodrigo.yanez.s@unab.cl; 3 School of Medicine, Universidad Espíritu Santo, Samborondón 092301, Ecuador; 4 Escuela de Entrenador en Actividad Física y Deporte, Facultad de Ciencias Humanas, Universidad Bernardo O’Higgins, Santiago 8370993, Chile; 5 Escuela de Kinesiología, Facultad de Salud, Universidad Santo Tomás, Talca 3460000, Chile; yenyconchaci@santotomas.cl (Y.C.-C.); eguzmanm@santotomas.cl (E.G.-M.); 6 Vicerrectoría de Investigación e Innovación, Universidad Arturo Prat, Iquique 1100000, Chile; 7 Escuela de Fonoaudiología, Facultad de Ciencias de la Salud, Universidad Autónoma de Chile, Talca 3460000, Chile; 8 Laboratorio de Evaluación y Cuidado del Estado Nutricio, División de Ciencias de la Salud, Centro Universitario de Tonalá, Universidad de Guadalajara, Tonalá 45425, Jalisco, Mexico; carlos.amante@academicos.udg.mx; 9 Red Iberoamericana de Investigadores en Antropometría Aplicada, 04120 Almería, Spain; 10 Marieb College of Health and Human Services, Florida Gulf Coast University, Fort Myers, FL 33965, USA; ljudge@fgcu.edu; 11 Escuela de Ciencias de la Actividad Física, el Deporte y la Salud, Universidad de Santiago de Chile (USACH), Santiago 9170022, Chile; claudio.farias.v@usach.cl; 12 Sports Coach Career, School of Education, Universidad Viña del Mar, Viña del Mar 2520000, Chile; 13 Escuela de Nutrición y Dietética, Facultad de Salud, Universidad Santo Tomás, Talca 3460000, Chile; 14 Department of Physical Activity Sciences, Universidad de Los Lagos, Osorno 5290000, Chile; jordan.hernandez@ulagos.cl; 15 Department of Education, Faculty of Humanities, Universidad de La Serena, La Serena 1700000, Chile

**Keywords:** machine learning, functional impairment, screening, gradient boosting, anthropometry, physical performance, older adults, Mexico, MHAS, random forest

## Abstract

**Background:** Slow gait, weak handgrip strength, and impaired balance are common functional deficits in aging populations that predict adverse health outcomes. Accurate, scalable prediction tools are needed for community-based screening in low-resource settings. **Objectives:** To benchmark nine machine learning (ML) classifiers for predicting the presence of ≥1 functional deficit using cardiometabolic and anthropometric predictors; to assess calibration, generalization, and clinical utility; to quantify the incremental value of these predictors over age and sex; and to characterize the stability of SHAP-based feature importance. **Methods:** We analyzed cross-sectional data from the Mexican Health and Aging Study 2012 (MHAS 2012) biomarker sub-study (N = 1961; mean age 61.9 ± 10.5 years; 59.8% women). The outcome was ≥1 of slow gait (<0.8 m/s), weak handgrip (EWGSOP2 sex-specific cut-points), or inability to sustain a 10-s one-leg stance (prevalence 78.7%). Twenty cardiometabolic and anthropometric predictors were used, excluding the functional measures used in outcome construction. Performance was estimated with nested, repeated stratified 10-fold cross-validation (5 × 10 outer folds; inner 5-fold hyperparameter tuning), with all preprocessing confined to training folds. **Results:** Logistic Regression (LR) achieved the highest discrimination (AUC-ROC 0.751, 95% CI 0.724–0.779), with good calibration (scaled Brier 0.125; calibration slope 0.96) and a small overfit gap (0.011); no classifier outperformed it (ET: AUC 0.745). An age–sex model alone reached AUC 0.734; all other predictors added ΔAUC = +0.018 (95% CI 0.004–0.031), almost entirely through anthropometry, whereas blood biomarkers and blood pressure added no measurable discrimination. SHAP identified age, sex, height, body weight, and BMI × Age as the most influential features; BMI ranked 12th of 20 (bootstrap 95% rank interval 8–20). **Conclusions:** Cardiometabolic and anthropometric predictors identify middle-aged and older Mexican people with ≥1 functional deficit with moderate accuracy (AUC ≈ 0.75), but most of the signal is carried by age, sex, and simple anthropometry. This reproducible, circularity- and leakage-free framework supports risk stratification rather than diagnosis, and requires external and temporal validation before clinical implementation.

## 1. Introduction

The increasing global burden of population ageing demands scalable and accurate tools to identify individuals at elevated risk of adverse health trajectories [1]. This challenge is compounded by declining birth rates, which progressively reduce the generational replacement base and intensify the pressure on healthcare systems that remain fragmented and lack sufficient geriatric specialists and multidisciplinary care teams [2,3]. Middle-aged and older adults face multiple preventable adverse trajectories, including falls, adverse drug events, malnutrition, social isolation, and elder abuse [4,5]. Caregiving systems are overburdened and understaffed [6], creating an urgent need for scalable risk stratification tools. One promising approach is biological age, defined as the rate at which an individual’s physiological systems deteriorate relative to chronological age. Chronological age alone treats all individuals born in the same year as equivalent, yet two people of identical calendar age may differ substantially in cardiovascular, metabolic, musculoskeletal, and immune function. Biological age captures this heterogeneity by integrating the cumulative wear and tear across multiple physiological systems [7], and has been shown to predict morbidity, functional decline, and mortality more accurately than calendar age alone [8,9]. However, its operationalization in epidemiological settings remains challenging, particularly in low- and middle-income countries and, more specifically, in Latin American settings, where access to molecular biomarkers (epigenetic clocks, telomere length) is limited [9]. In this context, functional deficits in gait speed, handgrip strength, and balance emerge as critical, measurable indicators of physiological decline in older adults [10,11].

Functional impairment is one observable correlate of accumulated physiological decline, characterized by loss of physical capacity and increased vulnerability to stressors [8,12]. It is, however, not specific to biological ageing: musculoskeletal, neurological, cardiorespiratory, and psychological conditions, acute injury, and physical inactivity can each produce deficits in gait, strength, or balance independently of the pace of biological ageing [12,13]. Functional performance measures are therefore best regarded as integrative indicators of current physical capacity rather than as direct readouts of biological age. With this caveat, the use of a composite functional deficit score derived from physical performance measures only allows for a measurable and scalable assessment of an individual’s current functional status [14,15]. Given that functional measures such as gait speed and handgrip strength have been individually validated as robust predictors of disability, morbidity, and mortality [16,17], with extensive meta-analytic evidence establishing precise clinical thresholds for pathological aging trajectories (e.g., gait speed deficits below 0.8 m/s and low handgrip strength associated with a 78–79% increased risk of functional decline and mortality), their integration represents a low-cost, scalable alternative for identifying adverse health outcomes [18,19]. Nevertheless, since no single measure captures the multidimensional nature of ageing, their combined predictive value, when integrated via machine learning (ML), has not been extensively evaluated in Latin American populations [12].

Addressing this gap requires analytical approaches capable of capturing the multidimensional nature of ageing; ML classifiers offer several advantages over traditional cut-off-based approaches: they capture non-linear relationships and feature interactions, handle correlated predictors without variable selection assumptions, and provide probabilistic outputs suitable for risk stratification [20,21]. Tree-based ensemble methods, particularly Gradient Boosting Machines and Random Forests, can outperform classical statistical models when strong non-linearities or high-order interactions are present, and sufficient data are available [22]. This advantage, however, has not been uniformly demonstrated: a systematic review of clinical prediction studies found no performance benefit of ML over logistic regression in comparisons at low risk of bias [23], and flexible algorithms generally require substantially larger samples than regression models to achieve stable performance [24]. Rigorous benchmarking across multiple classifiers and metrics therefore remains essential to prevent performance inflation from overfitting or evaluation bias [25].

A critical but often overlooked issue in this literature is circularity, a specific form of data leakage that arises when outcome construction relies on variables also used as model inputs [26]. In prediction of functional deficits specifically, the most accessible functional measures (gait speed, handgrip strength, balance) are simultaneously the most widely adopted criteria for constructing the outcome. Any classifier that receives these measures as inputs to predict an outcome defined by the same measures cannot distinguish between genuine biological learning and tautological pattern recognition [27]. Reported AUC values approaching 1.00 in such settings reflect this structural redundancy, not generalizable predictive performance [27]. A subtler form of leakage arises when data-dependent preprocessing (e.g., standardization or imputation) or hyperparameter selection is performed on the full dataset before cross-validation, which optimistically biases performance estimates; nested cross-validation, in which every such step is confined to the training folds, avoids this bias [28,29]. A further persistent methodological concern involves feature importance attribution. Mean Decrease in Impurity (MDI), the most commonly reported importance metric for tree-based classifiers, inflates the apparent contribution of continuous variables and suppresses low-cardinality features [30]. SHAP (SHapley Additive exPlanations) values derived from cooperative game theory provide theoretically consistent, model-agnostic, and individually interpretable importance estimates that overcome these limitations [31], although they describe how a model uses its inputs rather than causal effects, and their stability must be verified when predictors are correlated [32,33].

The Mexican Health and Aging Study 2012 (MHAS 2012) biomarker sub-study provides a suitable analytical opportunity. The MHAS is a nationally representative longitudinal cohort of Mexican adults aged 50 years and older and their spouses or partners [34], and its 2012 wave was the first to integrate venous blood-based biomarkers with objective anthropometric and physical performance measures in the same participants [35]. Importantly, the biomarker sub-study was not a random draw from the national sample: to contain costs and maximize standardization, it comprised the complete MHAS sample residing in four states (Sonora, Sinaloa, Jalisco, and Estado de México), selected to include a relatively poor, a highly urban, a high-migration, and a high-diabetes state [34,36]. The sub-study is therefore not nationally representative, and inferences from it pertain to community-dwelling middle-aged and older adults living in these four states. Its 2089 participants completed the full protocol, which included standardized blood pressure assessment, anthropometric measurements, performance tests (walking speed, handgrip strength, and one-leg balance), and venous blood collection in a single home visit by trained personnel from the National Institute of Public Health [34]. This integrated design enables a clean separation between the cardiometabolic predictor set and the functional outcome criteria, making MHAS 2012 particularly well suited for a methodologically rigorous prediction framework for functional deficits.

The aims of this study were (i) to benchmark nine ML classifiers for predicting the presence of ≥1 functional deficit (slow gait, weak grip, or impaired balance) using cardiometabolic and anthropometric predictors exclusively, with deliberate exclusion of functional performance measures from the predictor set, under a nested cross-validation framework free of preprocessing and tuning leakage; (ii) to assess model calibration, generalization, and potential clinical utility via multiple complementary metrics, including decision curve analysis; (iii) to quantify the incremental predictive value of anthropometric, haemodynamic, and blood-based markers over age and sex alone; and (iv) to characterize feature importance using SHAP values in place of MDI, together with the stability of these attributions, and to identify candidate cardiometabolic predictors that may inform early detection strategies for functional impairment in Latin American middle-aged and older populations. We hypothesized that (i) a cardiometabolic-anthropometric feature set excluding functional measures would yield moderate discriminative performance (AUC-ROC ≈ 0.65–0.75), (ii) Logistic Regression would match or exceed ensemble methods in calibration and generalization, and (iii) central adiposity indices and interaction terms would outweigh BMI in SHAP-based importance.

## 2. Materials and Methods

### 2.1. Study Design and Data Source

This is a secondary analysis of the MHAS 2012 biomarker sub-study, a cross-sectional data collection conducted in the complete MHAS sample residing in four Mexican states: Sonora, Sinaloa, Jalisco, and Estado de México [34,36]. Full sampling design, recruitment, and quality assurance procedures are described in detail elsewhere [34]. All participants provided written informed consent, and the study received ethical approval from the University of Wisconsin–Madison and the Instituto Nacional de Salud Pública, Mexico. The released sub-study file contained records with data for 2086 of the 2089 participants. Before modelling, the record-level linkage of every measurement block (anthropometry, blood pressure, physical performance, and laboratory determinations) to the household and person identifiers was verified through internal consistency checks, including the concordance between standing height and knee height (r = 0.91) and the expected sex differences in stature, handgrip strength, and haemoglobin. We excluded one record with an invalid age code (age_12 = 999) and 124 participants with missing data on ≥1 predictor (venous blood biomarkers unavailable, n = 86, of whom 4 also lacked anthropometry; incomplete anthropometry only, n = 38), yielding an analytic sample of 1961 participants with complete predictor data. Excluded participants were older than those included (66.3 ± 11.5 vs. 61.9 ± 10.5 years; *p* < 0.001) but did not differ in sex distribution (59.7% vs. 59.8% women; *p* = 1.00) or in the prevalence of ≥1 functional deficit (81.5% vs. 78.7%; *p* = 0.54). The primary analysis was a complete-case analysis; a sensitivity analysis with imputation of missing predictors is described in Section 2.8. Analyses were unweighted because the sub-study was not designed to yield nationally representative estimates.

### 2.2. Feature Engineering: Cardiometabolic and Anthropometric Predictors

Twenty predictors were constructed spanning four non-functional domains. Anthropometric domain: body mass index (BMI, kg/m^2^); waist-hip ratio (WHR); waist-to-height ratio (WtHR); absolute waist circumference (cm); hip circumference (cm); standing height (m); body weight (kg). Hemodynamic domain: mean systolic blood pressure (SBP, average of two readings); mean diastolic BP (DBP); pulse pressure (SBP − DBP, mmHg); mean arterial pressure (MAP = DBP + PP/3). Cardiometabolic biomarkers: C-reactive protein (CRP, mg/L); HDL-cholesterol (HDL-C, mg/dL); total cholesterol (mg/dL); thyroid-stimulating hormone (TSH, μIU/mL); 25-hydroxyvitamin D [25(OH)D, ng/mL]. Demographic covariates: age (years) and sex. Interaction terms: BMI × Age and WtHR × SBP, computed as products of the raw variables. Because CRP and TSH were strongly right-skewed (e.g., CRP range 0–207 mg/L), both were log-transformed [ln(x + 1)] to limit the leverage of extreme values; all other variables were used on their original scale. Pulse pressure and MAP are exact linear combinations of SBP and DBP, and several anthropometric indices are algebraically related (e.g., BMI with weight and height; WHR with waist and hip circumferences); these dependencies were retained to preserve the pre-specified feature set but were explicitly considered when interpreting feature attributions (Section 2.7). Functional measures (gait speed, handgrip strength, and balance) were deliberately excluded from the predictor set to prevent predictor-outcome circularity [37]. Continuous predictors were z-standardized within each training fold, and the fitted means and standard deviations were then applied to the corresponding validation fold (Section 2.5).

### 2.3. Composite Outcome: ≥1 Functional Deficit

The binary outcome was defined as the presence of ≥1 functional deficit across three physical performance measures: (i) slow gait speed, defined as completing a usual-pace 2.5-m timed walk in more than 3.125 s (<0.8 m/s) in the faster of two trials; because walking times were recorded in whole seconds, this criterion corresponds to a recorded time ≥ 4 s; (ii) weak handgrip strength, defined as a maximum handgrip strength across all available trials (up to two per hand) below the sex-specific cut-points of the revised European consensus on sarcopenia (EWGSOP2: <27 kg in men and <16 kg in women) [38]; and (iii) impaired postural balance, defined as inability to maintain an unassisted one-leg stance for 10 s on either leg [39]. Following the convention of standardized physical performance batteries, the best performance across attempts was used for each test [40]. Participants who attempted a test but could not complete it, who could not stand, or who were not tested because the interviewer judged the test unsafe were classified as having the corresponding deficit (balance: n = 103 unable on both legs; handgrip: n = 2), whereas refusals and missing or injured limbs were treated as missing for that component. Participants meeting one or more criteria were classified as having a functional deficit (FD = 1). Participants with no observed deficit and at least one missing component (n = 1) were classified as FD = 0; a sensitivity analysis restricted to participants with all three tests observed was also conducted.

Two features of this outcome require explicit justification. First, we used a single-criterion (≥1 deficit) threshold, consistent with a liberal operationalization of the Fried phenotype [41]. Each component is independently associated with disability, institutionalization, and mortality [18,19,39,42], so the presence of any deficit is clinically meaningful and, in community screening, may trigger comprehensive assessment or referral; this choice deliberately prioritizes sensitivity. The resulting outcome captures any functional limitation rather than the multisystem decline that characterizes frailty (≥3 criteria) and, given its high prevalence, the clinically less frequent class in this population is the absence of any deficit. To evaluate a more specific phenotype, all analyses were repeated for ≥2 deficits and for each component separately (Section 2.8). Second, the class imbalance implied by this prevalence affects performance assessment: precision–recall metrics, the Brier score, and all threshold-dependent metrics depend on prevalence, whereas the AUC-ROC does not [43]. We therefore report average precision for both classes against their respective baselines, the Brier score scaled against a non-informative model that assigns the training-fold prevalence to every participant, and threshold-dependent metrics at a threshold equal to the training-fold prevalence. Resampling (over- or undersampling) was not applied because it distorts predicted probabilities and calibration [44]; class weighting was evaluated in a sensitivity analysis.

### 2.4. Classifiers and Hyperparameter Configuration

Nine classifiers spanning five algorithm families were evaluated: (i) linear models: L2-penalized Logistic Regression (LR) and a penalized spline Logistic Regression (Spline LR), in which each continuous predictor was expanded into a cubic B-spline basis with four knots and linear tail extrapolation, allowing smooth non-linear effects analogous to restricted cubic splines [45]; (ii) tree-based ensembles: AdaBoost, Gradient Boosting (GB), Random Forest (RF), and Extra Trees (ET); (iii) probabilistic: Gaussian Naïve Bayes (NB); (iv) instance-based: K-Nearest Neighbors (K-NN); and (v) non-parametric: Decision Tree (DT). Hyperparameters were not fixed a priori but selected within the inner loop of the nested cross-validation (Section 2.5) from the following search spaces: LR, inverse regularization strength C ∈ {10^−3^, …, 10^1^} (nine log-spaced values); Spline LR, C ∈ {10^−3^, …, 10^0^} (seven values); AdaBoost, number of estimators {50, 100, 200, 300} × learning rate {0.03, 0.1, 0.3, 1.0}; GB, number of estimators {100, 200, 300} × learning rate {0.01, 0.03, 0.1} × maximum depth {2, 3, 4} × subsample {0.7, 1.0} × minimum leaf size {10, 30}; RF and ET (300 trees each), maximum depth {3, 5, 8, unlimited} × minimum leaf size {5, 20, 50} × features per split {√p, 0.33p, 0.5p}; DT, maximum depth {2, 3, 4, 5, 6, 8} × minimum leaf size {10, 20, 30, 50, 100}; NB, variance smoothing {10^−9^, …, 10^−1^}; and K-NN, K ∈ {11, 21, 31, 51, 75, 101, 151} with uniform or distance weighting. Exhaustive grid search was used for LR, Spline LR, DT, NB, and K-NN, and randomized search (eight configurations per inner loop) for AdaBoost, GB, RF, and ET. All models were implemented in Python v3.11 with scikit-learn v1.9 [46].

### 2.5. Evaluation Framework

Model performance was estimated with nested cross-validation to prevent information leakage and selection-induced optimism [28,29]. The outer loop was a stratified 10-fold cross-validation repeated five times with different random partitions (50 outer folds; random seed = 42), each fold preserving the outcome prevalence. Within each outer training set, an inner stratified 5-fold cross-validation selected hyperparameters by minimizing the log-loss, a strictly proper scoring rule that rewards both discrimination and calibration. Every data-dependent step (z-standardization, spline basis construction, imputation in the corresponding sensitivity analysis, and hyperparameter selection) was implemented within a single pipeline fitted exclusively on the training data of each loop, and each outer validation fold was used only once to evaluate the model refit on its training set. Metrics computed per outer fold and averaged were AUC-ROC (primary endpoint); average precision (AP) for the deficit and the no-deficit classes; Brier score and scaled Brier score (1 − Brier/Brier_null_); log-loss; sensitivity, specificity, balanced accuracy, F1 score, and Matthews Correlation Coefficient (MCC) [47] at a probability threshold equal to the training-fold prevalence; and the overfit gap (training AUC − validation AUC). Calibration intercept (calibration-in-the-large) and slope were estimated from pooled out-of-fold (OOF) predictions within each repetition and averaged [48,49]. Ninety-five percent confidence intervals (95% CI) for cross-validated means and paired comparisons of each classifier against LR (differences in AUC and Brier score across the same 50 outer folds) were obtained with the corrected resampled *t*-test, which accounts for the overlap of training sets across folds [50,51]; this replaces fold-level non-parametric tests, which ignore that dependence [52]. *p*-values were adjusted with the Holm method. OOF predicted probabilities were used to construct ROC, precision–recall, and calibration curves and to examine the observed prevalence across deciles of predicted risk. Potential clinical utility was assessed with decision curve analysis, which quantifies net benefit across threshold probabilities relative to the default strategies of assessing all or no individuals [53]. Discrimination and calibration were also examined in subgroups defined by sex and age (<65 vs. ≥65 years). Learning curves were derived for LR and the best-performing ensemble, and the minimum sample size required to develop a 20-parameter model was calculated using the criteria of Riley et al. (expected shrinkage ≥ 0.9, optimism in Nagelkerke R^2^ ≤ 0.05, and precise estimation of the overall risk) [54].

### 2.6. Incremental Predictive Value

To quantify the information contributed by each predictor domain beyond demographic structure, LR models of increasing complexity were evaluated on the same nested cross-validation partitions: age and sex alone; age, sex, and anthropometric indices; plus haemodynamic measures; plus blood biomarkers; and the full 20-predictor model including interaction terms. Because collinearity among anthropometric indices precludes a clean comparison of their SHAP values, the pre-specified hypothesis on adiposity indices was additionally examined by comparing age–sex models augmented with a single index (BMI, WHR, WtHR, or waist circumference). Differences in AUC relative to the age–sex model were tested with the corrected resampled *t*-test.

### 2.7. Model Explainability with SHAP Values

SHAP values [31] were computed for an LR model fitted on the full analytic sample (N = 1961). Because the cross-validated log-loss of LR was virtually flat over several orders of magnitude of C, many models with equivalent predictive performance but different allocations of credit among collinear predictors were available [55]; the weakly penalized optimum tends to assign large, offsetting coefficients to correlated predictors, which makes individual attributions unstable. For explanation, we therefore used the most strongly regularized model within one standard error of the minimum cross-validated log-loss (one-standard-error rule; C = 0.01) [56], which exploits the grouping effect of ridge penalization to distribute credit among correlated predictors while retaining equivalent discrimination. For a linear model with an interventional (independent) background distribution, SHAP values have the exact closed form φ_ij_ = β_j_(x_ij_ − E[x_j_]) on the standardized scale; we therefore computed them exactly, using the full sample as background, which removes the Monte Carlo error of sampled maskers. SHAP values quantify the additive contribution of each feature to each individual’s predicted log-odds of functional deficit. Summary visualizations use beeswarm plots: each point represents one participant; x-position encodes SHAP magnitude and direction; point color encodes the feature value (red = high, blue = low). Mean absolute SHAP (mean |SHAP|) is reported as the primary feature ranking statistic, replacing MDI throughout. Because SHAP values describe how a fitted model uses its inputs rather than causal effects, and because attributions among correlated or algebraically dependent features (e.g., SBP, DBP, pulse pressure, and MAP) are not uniquely identifiable [32,33], their stability was assessed in three ways: (i) 1000 bootstrap refits of the model, from which 95% intervals for mean |SHAP| and for each feature’s rank were obtained; (ii) agreement of feature rankings across the 50 outer-fold models, summarized with Kendall’s coefficient of concordance (W); and (iii) domain-level (grouped) SHAP values, obtained by summing the SHAP values of all features within a domain for each participant, which are invariant to how credit is shared among collinear features within that domain. Individual-level prediction instability was quantified as the mean absolute difference between each participant’s predicted risk from the bootstrap models and from the full-sample model [57].

### 2.8. Sensitivity Analyses and Reporting

The primary analysis was repeated for LR under the following alternative specifications: (i) the original operationalization of the outcome (first walking trial, right-leg stance only, a sex-invariant handgrip cut-point of 20 kg, and inability to perform a test coded as no deficit); (ii) ≥2 functional deficits; (iii–v) each functional component separately; (vi) a sex-invariant handgrip cut-point of 20 kg; (vii) participants with all three tests observed; (viii) class-weighted LR; (ix) untransformed CRP and TSH; (x) participants aged ≥50 years, the MHAS target population; and (xi) chained-equations imputation of missing predictors in all 2085 participants with a valid age, fitted within each training fold [58]. Reporting follows the TRIPOD + AI statement [59].

### 2.9. Use of Generative Artificial Intelligence

Generative artificial intelligence (GenAI) was used as an auxiliary tool during the development and refinement of Python code for the machine learning analyses, including model implementation, evaluation procedures, and data visualisation. The AI tool was used to assist with coding, troubleshooting, and methodological clarification. All code was executed and its outputs were reviewed and verified by the authors. The authors independently assessed the analytical procedures, interpreted the results, and take full responsibility for the accuracy, integrity, and reproducibility of the analyses and the conclusions drawn from them.

## 3. Results

### 3.1. Participant Characteristics

The final analytic sample consisted of 1961 participants (mean age 61.9 ± 10.5 years; 59.8% women), as summarized in Table 1. The prevalence of ≥1 functional deficit was 78.7% and was higher in women than in men (84.2% vs. 70.4%; *p* < 0.001). Slow gait was the most frequent component (70.4%), followed by impaired balance (35.0%) and weak handgrip strength (18.0%); 35.8% of participants had ≥2 deficits, and 8.7% had all three. Participants with a deficit were older, shorter, and had higher waist-to-height ratio, pulse pressure, and CRP, and lower 25(OH)D than those without a deficit (Table 1). The sample exhibited high levels of overweight and central adiposity (mean BMI 28.9 kg/m^2^; mean WtHR 0.63), and the median CRP was 2.5 mg/L.

### 3.2. Classifier Performance and Generalization

**Figure 1:**
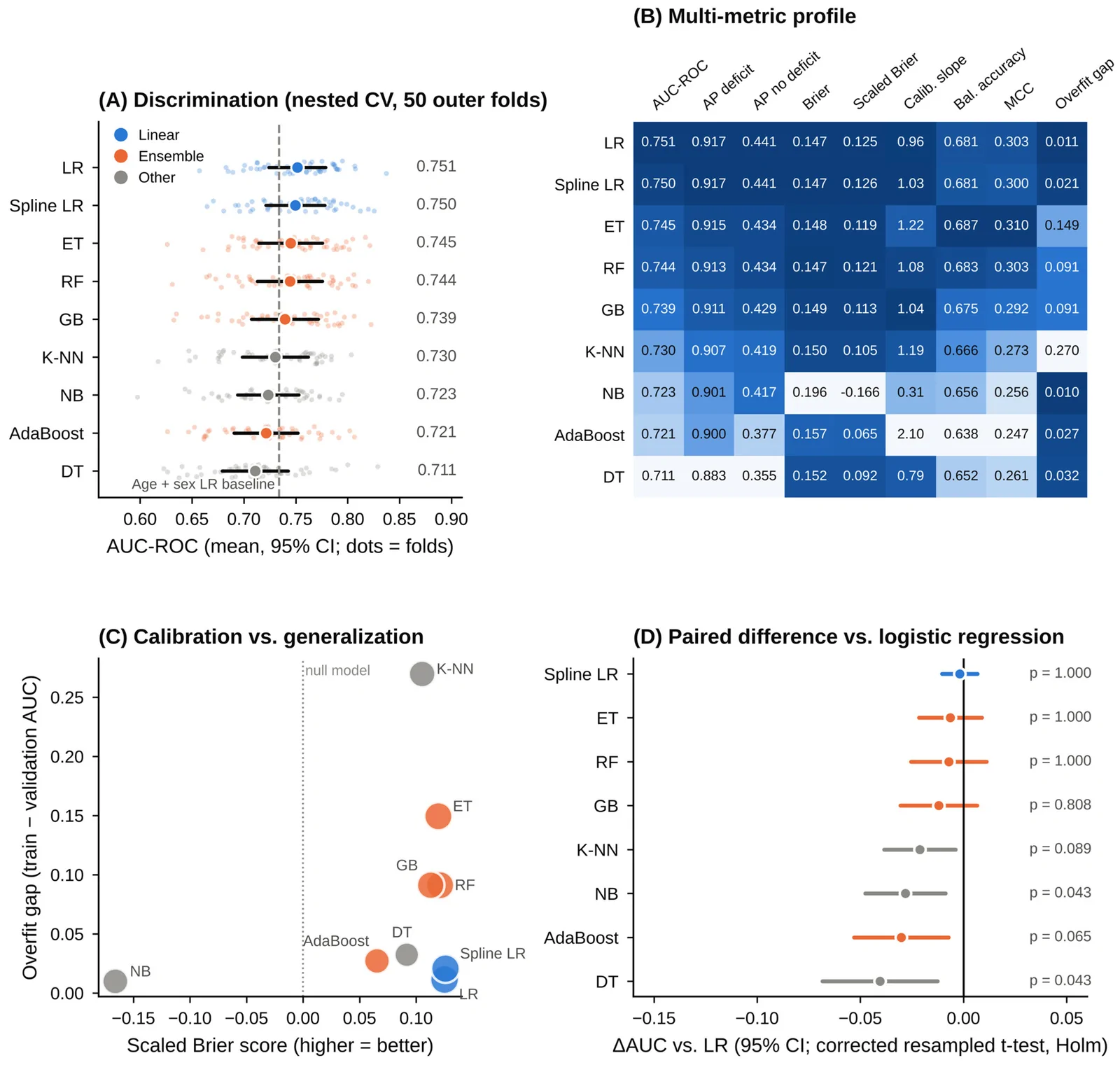
Classifier performance under nested cross-validation (N = 1961; 5 × 10 outer folds). (**A**) AUC-ROC for each classifier: small dots are the 50 outer-fold estimates, large markers are the mean, and horizontal bars are the 95% CI (corrected resampled *t*-test); the dashed line marks the age–sex Logistic Regression baseline. (**B**) Multi-metric heatmap; colours are normalized column-wise so that darker blue denotes relatively better performance (for calibration slope, closeness to 1), and cell values show the raw metric. (**C**) Calibration (scaled Brier score) versus generalization (overfit gap); marker area is proportional to AUC-ROC. (**D**) Paired differences in AUC-ROC relative to Logistic Regression across the same 50 folds (95% CI; Holm-adjusted *p*-values). Colours denote algorithm family (blue = linear; orange = tree ensembles; grey = other). AP = average precision; AUC = area under the receiver-operating characteristic curve; DT = Decision Tree; ET = Extra Trees; GB = Gradient Boosting; K-NN = K-Nearest Neighbors; LR = Logistic Regression; MCC = Matthews correlation coefficient; NB = Naïve Bayes; RF = Random Forest.

Under nested cross-validation, LR achieved the highest discriminative performance (AUC-ROC 0.751; 95% CI 0.724–0.779), closely followed by Spline LR (0.750) and ET (0.745) (Table 2; Figure 1A). LR also showed the most favourable overall profile, combining this discrimination with a Brier score (0.147; scaled Brier 0.125) virtually identical to the best value (Spline LR, 0.147; 0.126), near-ideal calibration (intercept 0.00; slope 0.96), and one of the smallest overfit gaps (0.011). The tree ensembles reached AUC values of 0.721–0.745, with overfit gaps of up to 0.149. In paired comparisons over the same 50 outer folds, no classifier significantly outperformed LR; 2 of the eight comparators had significantly lower AUC after Holm adjustment, and the remaining differences were small (ΔAUC between −0.030 and −0.002) (Figure 1D). Differences in Brier score were also small in absolute terms (≤0.002 for the five best classifiers), and should be interpreted alongside the scaled Brier score, which shows that all models explained only a modest fraction of the outcome variance beyond the non-informative prevalence model (scaled Brier ≤ 0.126). At the prevalence threshold, LR had a sensitivity of 0.666, a specificity of 0.697, a balanced accuracy of 0.681, and an MCC of 0.303. Figure 1B provides a multi-metric heatmap, and Figure 1C provides the calibration-versus-generalization profile.

### 3.3. ROC, Precision–Recall, Calibration, and Clinical Utility

**Figure 2:**
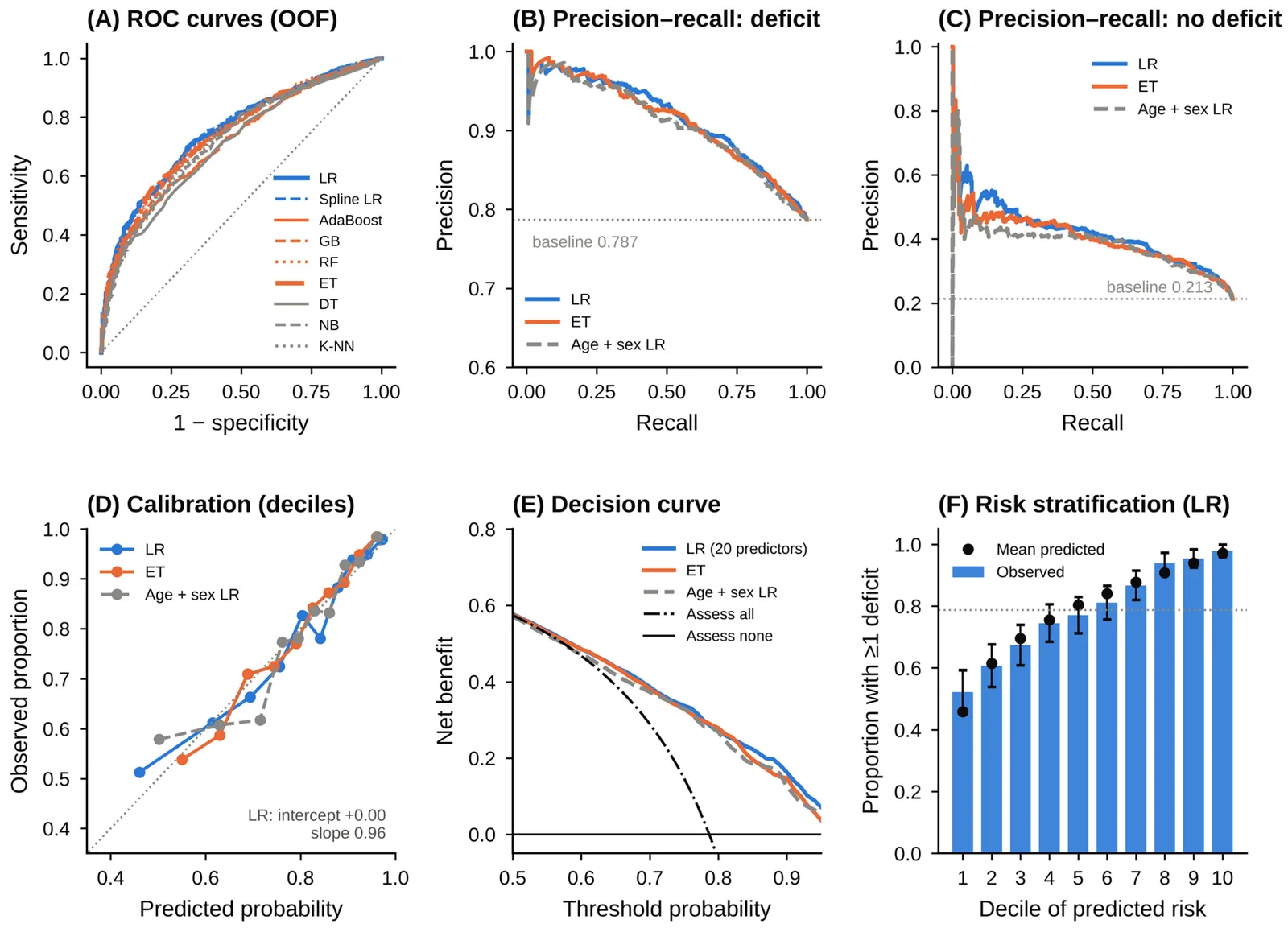
Discrimination, calibration, and clinical utility based on out-of-fold (OOF) predictions (N = 1961). (**A**) ROC curves for the nine classifiers (pooled OOF predictions of the first repetition; fold-averaged AUC values are given in Table 2). (**B**,**C**) Precision–recall curves for the deficit class (**B**) and the no-deficit class (**C**) for Logistic Regression (LR), ET, and the age–sex LR model; dotted lines indicate the corresponding class prevalence (baseline); fold-averaged average precision values are given in Table 2. (**D**) Calibration curves by decile of predicted risk; dotted diagonal = perfect calibration. (**E**) Decision curves showing net benefit across threshold probabilities compared with assessing all or no individuals. (**F**) Observed prevalence of ≥1 functional deficit (bars with 95% CI) and mean predicted risk (dots) by decile of LR-predicted risk (OOF predictions averaged over five repetitions); dotted line = overall prevalence. AP = average precision; ROC = receiver-operating characteristic.

Out-of-fold ROC curves overlapped substantially across the best-performing classifiers (Figure 2A). The average precision for the deficit class (LR, 0.917) exceeded its baseline of 0.787 only moderately (Figure 2B), whereas the average precision for the less frequent no-deficit class (0.441) was approximately twice its baseline of 0.213 (Figure 2C), indicating that the model’s main informational value lies in identifying individuals unlikely to have any deficit. Reliability diagrams confirmed that LR produced well-calibrated probability estimates across the risk spectrum (Figure 2D). In decision curve analysis, LR provided a meaningful net benefit (≥0.02) over the default strategy of assessing all individuals only at threshold probabilities ≥ 0.61, which correspond to settings in which an unnecessary assessment is considered at least 1.6 times as costly as a missed deficit; across thresholds of 0.50–0.90, its net benefit differed from that of the age–sex model by at most 0.038 (Figure 2E). Observed prevalence increased monotonically across deciles of predicted risk, from 52.3% in the lowest to 98.0% in the highest decile; the 20% of participants with the lowest predicted risk contained 40.9% of all participants without a deficit (Figure 2F).

### 3.4. SHAP Feature Importance and Attribution Stability

**Figure 3:**
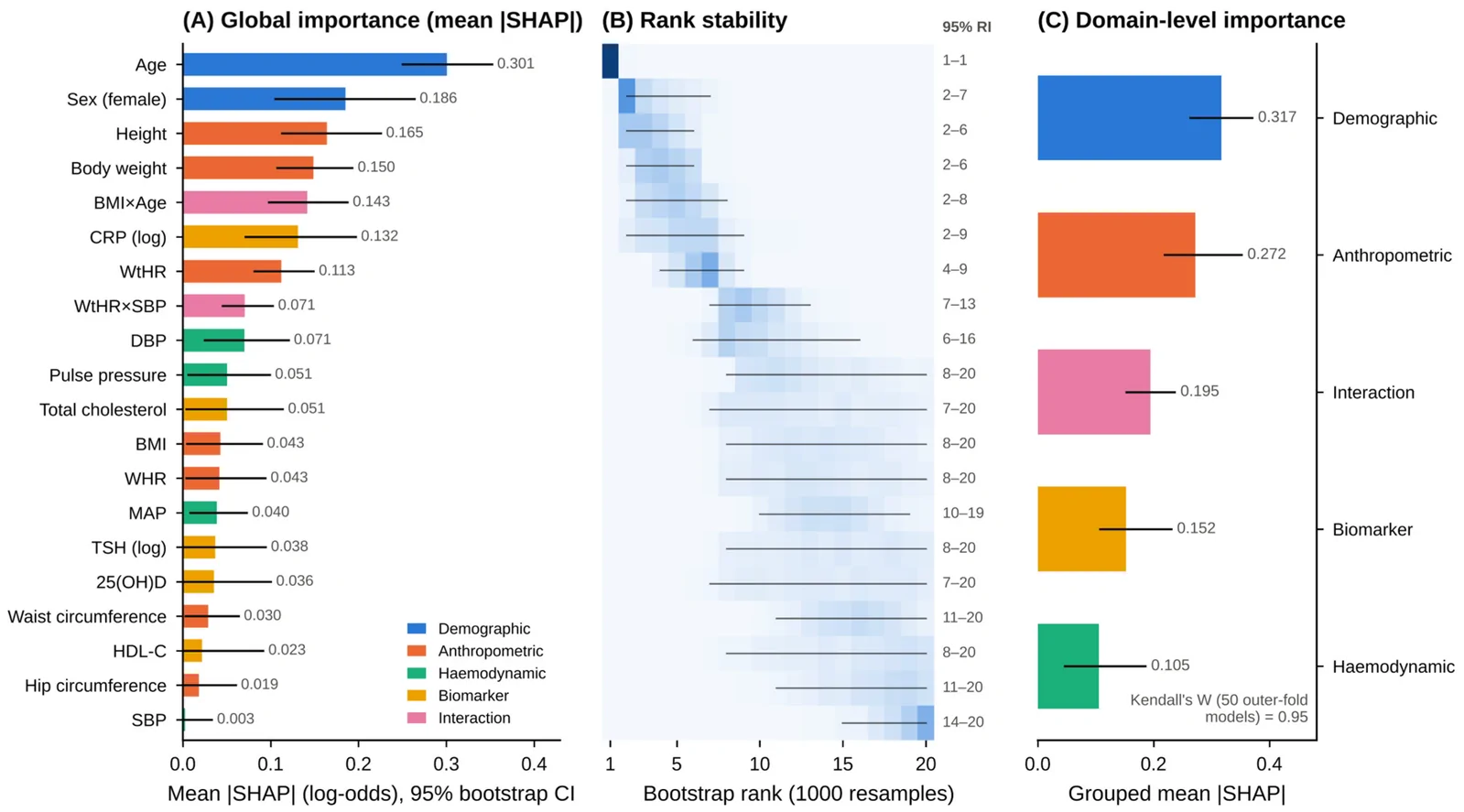
SHAP feature importance and attribution stability for the explanatory Logistic Regression model (one-standard-error penalty; N = 1961). (**A**) Mean absolute SHAP value of all 20 predictors with 95% bootstrap intervals (1000 resamples); colours denote predictor domain. (**B**) Bootstrap distribution of each feature’s importance rank (darker = more frequent), with 95% rank intervals on the right; features ordered as in (**A**). (**C**) Domain-level importance obtained by summing SHAP values within each domain, with 95% bootstrap intervals; Kendall’s W summarizes the concordance of rankings across the 50 outer-fold models. SHAP values were computed exactly for the linear model and replace Mean Decrease in Impurity (MDI) throughout. SHAP = SHapley Additive exPlanations.

**Figure 4:**
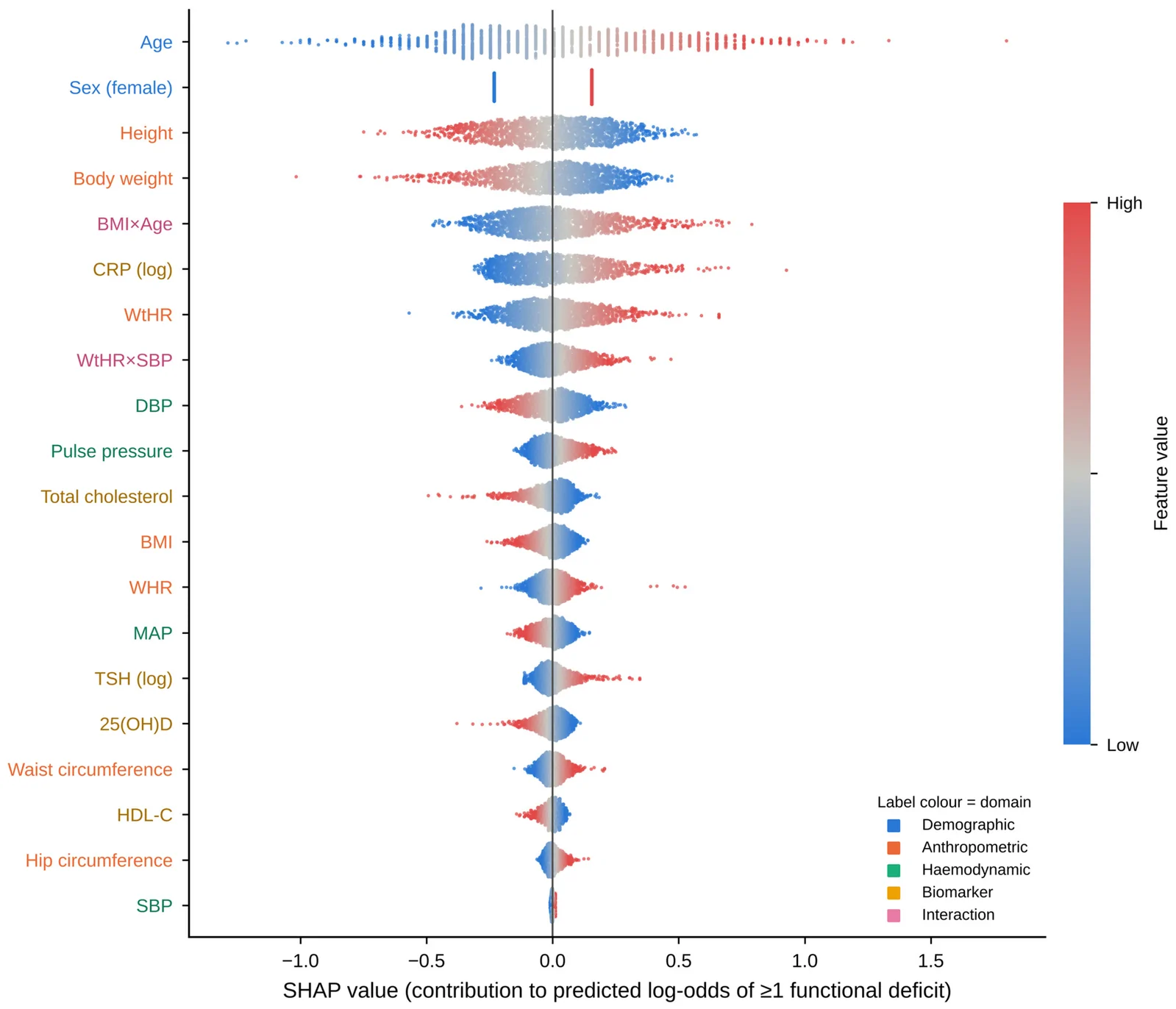
SHAP beeswarm plot for the explanatory Logistic Regression model (N = 1961). Each point represents one participant; the x-axis shows the SHAP value (positive values increase the predicted log-odds of having ≥1 functional deficit; negative values decrease it); point colour encodes the feature value (red = high, blue = low; for sex, red = female). Label colours indicate the predictor domain, and features are ordered by mean |SHAP|. Attributions are predictive rather than causal and are not uniquely identifiable among algebraically related features (Figure 3B). 25(OH)D = 25-hydroxyvitamin D; BMI = body mass index; CRP = C-reactive protein; DBP = diastolic blood pressure; HDL-C = high-density lipoprotein cholesterol; MAP = mean arterial pressure; SBP = systolic blood pressure; TSH = thyroid-stimulating hormone; WHR = waist-hip ratio; WtHR = waist-to-height ratio.

In the explanatory LR model (C = 0.01; nested cross-validated AUC 0.749, equivalent to that of the prediction model), SHAP analysis identified age (mean |SHAP| = 0.301) as the most influential predictor, followed by sex (0.186), height (0.165), body weight (0.150), and BMI × Age (0.143) (Figure 3A). Women showed systematically higher predicted log-odds of deficit than men after adjustment for the other predictors, and shorter stature was associated with higher predicted risk (Figure 4). The rankings of the dominant predictors were stable across bootstrap resamples (95% rank intervals: age 1–1; sex 2–7; height 2–6), whereas lower-ranked features (DBP, pulse pressure, total cholesterol, BMI, WHR, TSH, 25(OH)D, and HDL-C) had rank intervals spanning ten or more positions, reflecting small attributions and the sharing of credit among correlated variables (Figure 3B); overall agreement of rankings across the 50 outer-fold models was high (Kendall’s W = 0.95).

BMI ranked 12th of 20 (mean |SHAP| = 0.043; bootstrap 95% rank interval 8–20), and the BMI × Age and WtHR × SBP interaction terms ranked 5th and 8th, respectively. At the domain level, which is robust to collinearity within domains, demographic variables carried the largest share of importance (grouped mean |SHAP| = 0.317), followed by the anthropometric (0.272), interaction (0.195), blood biomarker (0.152), and haemodynamic (0.105) domains (Figure 3C). Among laboratory biomarkers, higher CRP was the only marker with a consistently non-negligible attribution (rank 6th; 95% rank interval 2–9); lower 25(OH)D (rank 16th) was associated with slightly higher predicted risk, and HDL-C, total cholesterol, and TSH contributed minimally (Figure 4). These attributions describe predictive associations within the fitted model and do not imply causal effects. 

### 3.5. Model Diagnostics and Incremental Predictive Value

**Figure 5:**
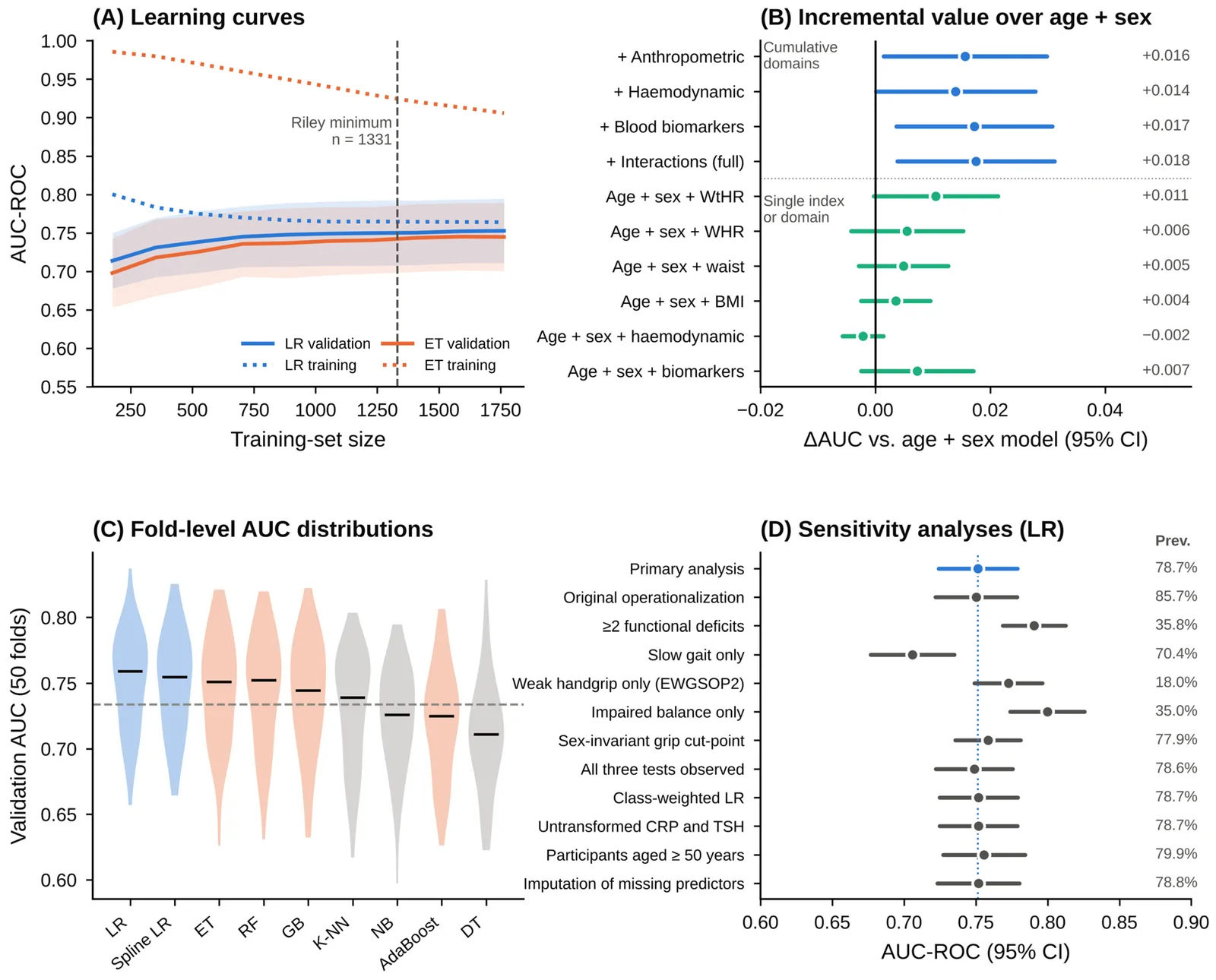
Model diagnostics, incremental value, and sensitivity analyses. (**A**) Learning curves for Logistic Regression (LR) and ET (solid lines = validation AUC; dotted lines = training AUC; shaded bands = ±1 SD over 3 × 10-fold cross-validation); the dashed vertical line marks the minimum sample size according to Riley et al. (**B**) Change in AUC relative to the age–sex LR model for cumulative domain additions (blue) and for single indices or domains added to age and sex (green), with 95% CI. (**C**) Fold-level AUC distributions across the 50 outer folds (horizontal lines = medians; dashed line = age–sex baseline). (**D**) AUC (95% CI) of LR under alternative outcome definitions and analytic choices (Table 4); the prevalence of the modelled outcome is shown on the right. AUC = area under the receiver-operating characteristic curve.

Learning curves showed that the validation AUC of LR approached a plateau at approximately 1100 training samples, with converging training and validation curves, whereas ET retained a larger training–validation gap (Figure 5A). The minimum sample size required to develop a 20-parameter model according to the criteria of Riley et al. was 1331, below the available sample. These diagnostics indicate stable internal estimation for LR but do not establish generalizability to other populations, which requires external validation. In the incremental-value analysis (Table 3; Figure 5B), age and sex alone achieved an AUC of 0.734 (95% CI 0.707–0.761). Adding anthropometric indices increased the AUC (ΔAUC = +0.016; 95% CI 0.001–0.030); further addition of haemodynamic measures, blood biomarkers, and interaction terms did not improve discrimination appreciably (full model ΔAUC = +0.018; 95% CI 0.004–0.031). Among single adiposity indices added to age and sex, WtHR produced the largest gain (ΔAUC = +0.011), followed by WHR (+0.006), waist circumference (+0.005), and BMI (+0.004); none of these differences was statistically significant. Fold-level AUC distributions are shown in Figure 5C.

**Table 3:** Incremental predictive value of predictor domains over age and sex (Logistic Regression; nested cross-validation, 5 × 10 outer folds; N = 1961).

Logistic Regression Model	Predictors (n)	AUC-ROC (95% CI)	ΔAUC vs. Age + Sex (95% CI)	*p*	Scaled Brier	AP (No Deficit)
Nested domain additions						
Age + sex	2	0.734 (0.707–0.761)	Reference	—	0.103	0.406
+Anthropometric	9	0.750 (0.722–0.777)	+0.016 (+0.001, +0.030)	0.032	0.124	0.437
+Haemodynamic	13	0.748 (0.720–0.775)	+0.014 (+0.000, +0.028)	0.048	0.123	0.437
+Blood biomarkers	18	0.751 (0.724–0.778)	+0.017 (+0.004, +0.031)	0.014	0.125	0.441
+Interaction terms (full model)	20	0.751 (0.724–0.779)	+0.018 (+0.004, +0.031)	0.013	0.125	0.441
Single index or domain added to age + sex						
Age + sex + BMI	3	0.737 (0.710–0.765)	+0.004 (−0.002, +0.010)	0.244	0.106	0.414
Age + sex + WHR	3	0.739 (0.714–0.765)	+0.006 (−0.004, +0.015)	0.261	0.112	0.422
Age + sex + WtHR	3	0.744 (0.718–0.771)	+0.011 (−0.000, +0.021)	0.056	0.116	0.425
Age + sex + waist	3	0.739 (0.713–0.765)	+0.005 (−0.003, +0.013)	0.211	0.109	0.417
Age + sex + haemodynamic	6	0.732 (0.705–0.759)	−0.002 (−0.006, +0.001)	0.224	0.101	0.408
Age + sex + biomarkers	7	0.741 (0.715–0.768)	+0.007 (−0.002, +0.017)	0.140	0.111	0.419
Single domains alone						
Age only	1	0.677 (0.648–0.707)	−0.057 (−0.076, −0.037)	<0.001	0.058	0.338
Sex only	1	0.599 (0.574–0.623)	−0.135 (−0.160, −0.110)	<0.001	0.027	0.261
Anthropometric only	7	0.701 (0.671–0.732)	−0.032 (−0.062, −0.003)	0.033	0.082	0.395
Biomarkers only	5	0.601 (0.568–0.635)	−0.133 (−0.172, −0.094)	<0.001	0.022	0.312

All models were evaluated on identical outer folds with C tuned in the inner loop. Nested additions are cumulative. Confidence intervals and *p*-values are from the corrected resampled *t*-test (not adjusted for multiplicity). Anthropometric = BMI, WHR, WtHR, waist and hip circumferences, height, and weight; haemodynamic = SBP, DBP, pulse pressure, and MAP; biomarkers = CRP, HDL-C, total cholesterol, TSH, and 25(OH)D. AP (no deficit) baseline = 0.213.

**Table 4:** Sensitivity analyses for Logistic Regression under alternative outcome definitions and analytic choices (nested cross-validation, 5 × 10 outer folds).

Analysis	N	Prevalence (%)	AUC-ROC (95% CI)	AP (No Deficit) (Baseline)	Scaled Brier	Calib. Intercept	Calib. Slope
Primary analysis	1961	78.7	0.751 (0.724–0.779)	0.441 (0.213)	0.125	0.00	0.96
Original operationalization ^1^	1961	85.7	0.750 (0.722–0.779)	0.347 (0.143)	0.096	0.00	0.92
≥2 functional deficits	1961	35.8	0.791 (0.769–0.812)	0.850 (0.642)	0.262	0.00	0.98
Slow gait only	1959	70.4	0.706 (0.677–0.735)	0.488 (0.296)	0.101	0.00	0.98
Weak handgrip only (EWGSOP2)	1961	18.0	0.773 (0.749–0.796)	0.932 (0.820)	0.168	0.00	0.97
Impaired balance only	1953	35.0	0.800 (0.774–0.826)	0.864 (0.650)	0.268	0.00	0.98
Sex-invariant handgrip cut-point (<20 kg)	1961	77.9	0.758 (0.736–0.781)	0.458 (0.221)	0.134	0.00	0.95
All three tests observed	1952	78.6	0.749 (0.722–0.776)	0.439 (0.214)	0.123	0.00	0.95
Class-weighted LR	1961	78.7	0.752 (0.725–0.779)	0.440 (0.213)	−0.220	+1.31	0.90
Untransformed CRP and TSH	1961	78.7	0.752 (0.725–0.779)	0.439 (0.213)	0.126	0.00	0.97
Participants aged ≥ 50 years	1812	79.9	0.756 (0.727–0.784)	0.435 (0.201)	0.127	0.00	0.97
Imputation of missing predictors ^2^	2085	78.8	0.752 (0.723–0.780)	0.441 (0.212)	0.126	0.00	0.95

^1^ First walking trial, right-leg stance only, sex-invariant 20-kg handgrip cut-point, and inability to perform a test coded as no deficit. ^2^ Chained-equations imputation fitted within each training fold, in all participants with a valid age. Calib. = calibration. For single-component outcomes, the modelled outcome is the corresponding deficit and the baseline refers to its absence. Confidence intervals are from the corrected resampled *t*-test. AP = average precision; LR = Logistic Regression.

### 3.6. Sensitivity and Subgroup Analyses

**Figure 6:**
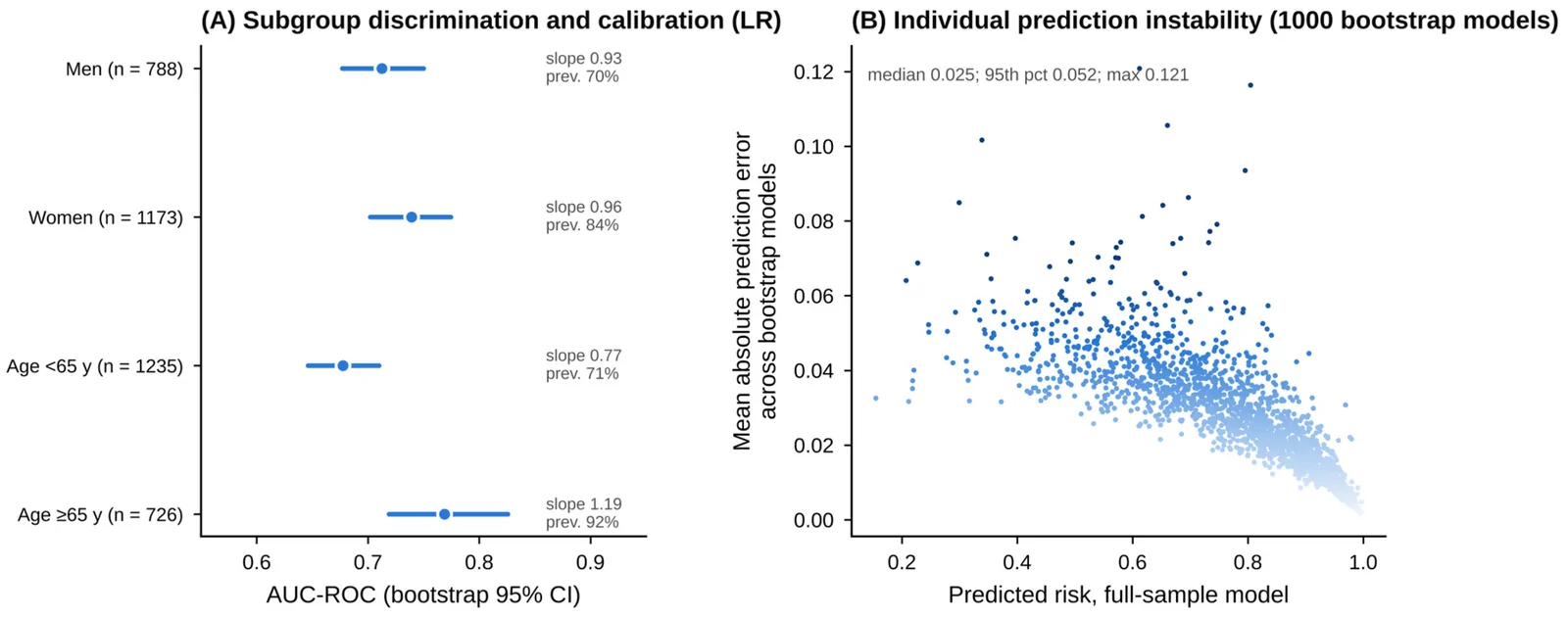
Subgroup performance and individual prediction stability of Logistic Regression (N = 1961). (**A**) AUC-ROC (bootstrap 95% CI), calibration slope, and prevalence by sex and age group, based on out-of-fold predictions. (**B**) Mean absolute difference between each participant’s predicted risk from 1000 bootstrap refits of the prediction model and from the full-sample model, plotted against the full-sample predicted risk. AUC = area under the receiver-operating characteristic curve.

Discrimination was robust to the analytic choices examined (Table 4; Figure 5D). Under the original operationalization of the outcome, prevalence rose to 85.7%, but the AUC was unchanged (0.750), whereas the precision for the no-deficit class and the scaled Brier score decreased, reflecting the smaller and noisier minority class. Discrimination was higher for ≥2 deficits (AUC 0.791; prevalence 35.8%) and for the individual components impaired balance (0.800) and weak handgrip (0.773) than for slow gait (0.706). Using a sex-invariant 20-kg handgrip cut-point (0.758), restricting the analysis to participants with all three tests observed (0.749), using untransformed CRP and TSH (0.752), restricting to participants aged ≥50 years (0.756), or imputing missing predictors (0.752; N = 2085) produced similar results. Class weighting left the AUC unchanged (0.752) but severely miscalibrated the predicted probabilities (calibration intercept +1.31; scaled Brier −0.22). In subgroup analyses based on OOF predictions, discrimination was lower within strata of sex and age than overall, as expected when stratifying on the two strongest predictors (AUC 0.712 in men and 0.739 in women; 0.677 in participants aged <65 years and 0.769 in those aged ≥65 years); calibration slopes ranged from 0.77 to 1.19, and risk was underestimated among participants aged ≥65 years (calibration intercept +0.30) (Figure 6A). In addition, individual predictions of the prediction model were stable across 1000 bootstrap refits (median mean absolute prediction error 0.025; 95th percentile 0.052; Figure 6B).

## 4. Discussion

This study presents a systematic, leakage-free ML benchmarking framework for predicting ≥1 functional deficit in Mexican middle-aged and older people that deliberately excludes functional performance measures from the predictor set, thereby eliminating predictor-outcome circularity. The principal findings are: (i) cardiometabolic and anthropometric predictors achieve moderate discrimination (AUC = 0.751) for identifying individuals with any functional deficit, without recourse to gait speed, handgrip strength, or balance; (ii) none of eight alternative classifiers, including flexible ensembles and a spline-based model, improved on penalized LR, which combined the highest discrimination with calibration and generalization comparable to the best alternatives in this sample; (iii) most of the predictive signal is carried by age and sex (AUC = 0.734), and the incremental contribution of the remaining predictors is small (ΔAUC = +0.018) and attributable almost entirely to inexpensive anthropometry, whereas blood pressure and venous blood biomarkers add no measurable discrimination; and (iv) SHAP-based importance is stable for the dominant predictors but not for individual collinear anthropometric and haemodynamic features, which should therefore be interpreted at the domain level. These findings support our first two pre-specified hypotheses; the third (predominance of central adiposity indices and interaction terms over BMI) received only weak support, as discussed below.

The AUC of 0.751 achieved without functional predictors represents discriminative performance that is unlikely to be inflated by circularity or by leakage from preprocessing and tuning. Published AUC values of 0.95–1.00 for frailty prediction frequently arise from structural circularity: when gait speed, handgrip strength, or balance appear in both the predictor set and the outcome definition, any classifier need only recognize the definitional redundancy to achieve ceiling performance [26,27]. The value reported here reflects the predictive signal available from blood biomarkers, anthropometry, and blood pressure in this sample. Its clinical utility, however, should be interpreted cautiously. Discrimination is moderate, and with a deficit prevalence of 78.7%, a strategy of assessing everyone already achieves a high positive predictive value; correspondingly, the average precision for the deficit class only modestly exceeded its baseline, and decision curve analysis showed a meaningful net benefit over assessing all individuals only at high threshold probabilities (≥0.61), which are unlikely to apply when performance tests are inexpensive and safe [53,60]. The model’s informational value lies mainly at the low-risk end of the distribution: the average precision for the no-deficit class was about twice its baseline, and observed prevalence ranged from 52.3% in the lowest to 98.0% in the highest decile of predicted risk. In practice, such a tool could at most help to prioritize who should undergo direct performance testing when testing capacity is limited; it cannot replace that testing, and it is not a diagnostic instrument. The good calibration of LR (scaled Brier 0.125; calibration slope 0.96) indicates that its probability estimates are reliable within this population, which is a prerequisite for risk stratification but not evidence of clinical benefit [48,61].

In this sample, LR offered the most favourable overall profile among the nine classifiers, combining the highest AUC with a near-optimal Brier score (0.147), near-ideal calibration slope (0.96), and a minimal overfit gap (0.011). Well-calibrated probability outputs are essential in clinical risk stratification: a model that assigns a probability of 0.80 of having a functional deficit should observe approximately 80% of those individuals to indeed have a deficit. The tree ensembles reached similar or lower AUC values with larger overfit gaps (up to 0.149), and none outperformed LR in paired comparisons. Nevertheless, the absolute differences in Brier score between the best classifiers were small, and in the absence of external validation, these results do not establish that LR is superior in general; they indicate only that additional model flexibility did not translate into better out-of-sample performance in these data. This pattern is consistent with evidence that regularized regression frequently matches complex ML methods in clinical prediction [23] and that flexible algorithms require larger samples to realize their potential [24]. The near-identical performance of Spline LR further suggests that non-linear effects of the continuous predictors contributed little beyond the linear terms. As argued in the calibration literature [48], flexible algorithms are particularly prone to miscalibration due to overfitting when the sample size is limited relative to model complexity, and discrimination alone is insufficient to guarantee reliable risk estimates [61]. Our class-weighting sensitivity analysis illustrates the same point: re-weighting left the AUC unchanged but severely degraded calibration (calibration intercept 1.31; scaled Brier −0.22), confirming that imbalance corrections are unnecessary and potentially harmful for risk estimation [44].

The SHAP analysis adds interpretability to the performance metrics, but its results describe how the model uses its inputs and should not be read as evidence of pathophysiological mechanisms [33]. The primacy of age and sex reflects the fundamental role of demographic ageing trajectories in functional vulnerability, consistent with epidemiological evidence across diverse populations [14]. The higher predicted risk among women is consistent with their higher prevalence of slow gait and impaired balance in this sample, despite the use of sex-specific handgrip cut-points, which prevented a sex difference in muscle strength from being built into the outcome definition. Within the anthropometric domain, shorter stature and higher WtHR were associated with higher predicted risk; however, because BMI, weight, height, waist, hip, WHR, and WtHR are algebraically related, the credit assigned to any single index depends on which correlated indices are present in the model, and individual rankings were correspondingly unstable. A design that avoids this problem is the comparison of age–sex models augmented with a single index: WtHR yielded the largest gain, followed by WHR, waist circumference, and BMI, but all differences were small and non-significant. Our third hypothesis is therefore only weakly supported: central adiposity indices, particularly WtHR, carried marginally more predictive information than BMI, but the data do not support strong claims about the superiority of central over total adiposity. Previous studies linking visceral adiposity and dynapenic or sarcopenic obesity to accelerated functional decline [62,63] provide a plausible context for these associations, but the present cross-sectional, predictive analysis cannot test them. Similarly, the interaction terms (BMI × Age and WtHR × SBP) did not improve discrimination, and their attributions cannot be separated from those of the main effects with which they are strongly correlated.

Among laboratory biomarkers, lower 25(OH)D and higher CRP were associated with higher predicted risk, directions consistent with the proposed roles of vitamin D deficiency in musculoskeletal deterioration [16] and of chronic low-grade inflammation (“inflammaging”) in physiological decline [64,65]. These associations must be interpreted with caution: apart from CRP, individual biomarkers received small and unstable attributions; adding blood biomarkers to age, sex, and anthropometry did not improve discrimination, and reverse causation is plausible (e.g., functional limitation may reduce outdoor activity and sun exposure, lowering 25(OH)D). A practically important implication is that, for identifying current functional deficits, the information obtainable from a venous blood draw was largely redundant once age, sex, and simple anthropometric measurements were known. The directional interpretability provided by SHAP, showing which feature values increase or decrease the predicted probability of functional deficit for individual participants, supports transparency of the model but does not by itself make it a decision-support tool.

### 4.1. Novelty and Practical Implications

Beyond its specific estimates, this study contributes four elements that, to our knowledge, have not previously been combined for functional deficit prediction in a Latin American cohort. First, it provides a fully nested, leakage-free benchmark in which outcome-defining measures, preprocessing, and hyperparameter selection are all kept out of the evaluation data, so that the reported AUC of approximately 0.75 can serve as a realistic reference value against which more optimistic, circularity-prone estimates can be judged. Second, it explicitly quantifies the incremental value of each predictor domain over age and sex, showing that blood-based cardiometabolic biomarkers—the most expensive and logistically demanding component of the data collection—added no measurable discrimination once age, sex, and anthropometry were known. This finding challenges the implicit assumption that multi-biomarker panels are needed to screen for current functional impairment and suggests that, in resource-limited community settings, investment in direct performance testing (which requires only a stopwatch, a dynamometer, and a few minutes) or in basic anthropometry is likely to be more efficient than venous blood collection for this purpose.

Third, it moves explainability from single, unqualified rankings to stability-aware interpretation, combining exact linear SHAP values with bootstrap rank intervals, cross-fold concordance, domain-level attributions, and individual-level prediction instability; this approach exposes which attributions are robust (age, sex, and the anthropometric domain) and which are artefacts of collinearity, and is directly transferable to other cohorts. Fourth, it shows how outcome operationalization (best versus first attempt, sex-specific versus sex-invariant handgrip cut-points, and coding of inability to perform a test) materially changes prevalence while leaving discrimination largely unchanged, which is relevant for harmonizing functional outcomes across ageing cohorts. From a practical standpoint, the framework and the accompanying analytic choices offer a reproducible template for evaluating low-cost screening strategies in ageing populations of low- and middle-income countries, and the calibrated LR model could be used, after external validation, to prioritize individuals for in-person functional assessment when testing capacity is scarce.

### 4.2. Limitations and Future Directions

This study has several limitations. First, our outcome definition (≥1 of three functional criteria) produces a 78.7% prevalence. We deliberately chose this liberal threshold to maximize sensitivity for community-based screening, where missing any functional deficit could have adverse consequences. However, such a permissive definition groups individuals with a single, possibly mild or transient limitation together with those with severe multidomain impairment, which may limit the model’s ability to identify individuals with clinically significant functional impairment and reduce the clinical specificity of a positive classification. Our model therefore predicts “any functional deficit” rather than the multisystem decline characteristic of the Fried frailty phenotype (≥3 criteria), and its outputs should be interpreted as screening for any functional impairment, not as a diagnostic tool for frailty. The higher discrimination observed for ≥2 deficits (AUC = 0.791) and for impaired balance alone (AUC = 0.800) suggests that more specific phenotypes are more predictable, and future work should develop and validate models for ≥2 and ≥3 criteria, which require separate estimation because of their different class distributions.

Second, the high prevalence implies that the no-deficit group is the minority class; although we did not apply resampling, which would have distorted calibration, threshold-dependent metrics depend on the chosen threshold and on prevalence and are therefore not directly transportable to populations with a different burden of functional impairment. Third, measurement characteristics of the MHAS protocol may have introduced misclassification: walking times were recorded in whole seconds over a short course, so that the 0.8 m/s threshold operates as a ≥4-s criterion; the one-leg stance test is demanding for older adults; and the EWGSOP2 handgrip cut-points were derived in European populations and may not be optimal for Mexican adults. Fourth, the cross-sectional design prevents causal inference, provides no information on the temporal ordering between predictors and outcome, and precludes prospective modelling of functional decline; longitudinal validation is an essential next step following the TRIPOD and TRIPOD + AI frameworks [59,66].

Fifth, the analytic sample was drawn from the complete MHAS sample of four Mexican states rather than from a nationally representative sample, and analyses were unweighted; generalizability to the national population and to other Latin American populations is therefore uncertain. Participants excluded because of missing predictors were older than those included, although an imputation-based sensitivity analysis yielded similar performance (AUC = 0.752). Sixth, the data were collected in 2012. Since then, Mexico has continued to experience rising obesity—from 32.4% of adults in 2012 to 36.9% in 2022 [67]—alongside persistently high levels of insufficient physical activity [68] and an ongoing dietary transition towards ultra-processed foods [69]. Such secular changes can shift both the distribution of predictors and their associations with functional outcomes (dataset shift), degrading the calibration and potentially the discrimination of models developed on older data [70,71]; in contemporary cohorts, adiposity-related and lifestyle predictors might carry greater importance than in our sample. Temporal validation in later MHAS waves, including the 2016 biomarker collection [35], and recalibration before any implementation are therefore necessary.

Seventh, although nested cross-validation provides nearly unbiased internal estimates and the learning curves suggest stable estimation for LR, internal validation does not guarantee transportability; external validation in independent, nationally representative samples such as ENSANUT or SABE is an essential requirement before the model can be recommended for routine use in clinical or community practice [72]. Finally, SHAP values were derived from a single model class, quantify predictive rather than causal contributions, and are not uniquely identifiable for collinear predictors; individual feature rankings should thus be interpreted with the reported uncertainty, preferably at the domain level.

## 5. Conclusions

This study demonstrates that a set of cardiometabolic and anthropometric predictors, deliberately excluding functional performance measures, can identify middle-aged and older Mexican adults with ≥1 functional deficit with moderate discrimination (AUC = 0.751) under a leakage-free, nested cross-validation framework. Logistic Regression provided the most robust model profile in this sample, combining the highest discrimination with good calibration (scaled Brier = 0.125; calibration slope = 0.96) and a minimal overfit gap (0.011); no flexible ML algorithm improved on it. SHAP explainability identified age, sex, and the anthropometric domain as the most influential and stable contributors, whereas BMI ranked 12th of 20 and blood biomarkers contributed little. Most of the discriminative signal was carried by age and sex, and the incremental value of the remaining predictors (ΔAUC = +0.018) came almost entirely from simple anthropometry. These results support the use of such models for risk stratification and prioritization rather than diagnosis, and suggest that venous blood biomarkers add little to the identification of current functional deficits. As this analysis was based on a regional sub-study collected in 2012, the findings should be considered preliminary and require external and temporal validation before clinical implementation.

## Figures and Tables

**Table 1:** Characteristics of the analytic sample of the MHAS 2012 biomarker sub-study (N = 1961), overall, by sex, and by functional deficit (FD) status.

Characteristic	Total (N = 1961)	Men (n = 788)	Women (n = 1173)	No FD (n = 418)	FD (n = 1543)	*p*
Demographic						
Age (years), mean ± SD	61.9 ± 10.5	63.4 ± 10.0	60.8 ± 10.7	56.8 ± 8.0	63.2 ± 10.7	<0.001
Age ≥ 65 years, n (%)	726 (37.0)	338 (42.9)	388 (33.1)	60 (14.4)	666 (43.2)	<0.001
Women, n (%)	1173 (59.8)	0 (0.0)	1173 (100.0)	185 (44.3)	988 (64.0)	<0.001
Anthropometric						
Height (m), mean ± SD	1.55 ± 0.10	1.63 ± 0.07	1.50 ± 0.07	1.59 ± 0.09	1.54 ± 0.09	<0.001
Body weight (kg), mean ± SD	69.7 ± 15.1	74.4 ± 14.7	66.5 ± 14.4	72.2 ± 14.9	69.0 ± 15.0	<0.001
BMI (kg/m^2^), mean ± SD	28.9 ± 5.3	27.9 ± 4.7	29.6 ± 5.6	28.4 ± 4.7	29.0 ± 5.4	0.015
Waist circumference (cm), mean ± SD	98.1 ± 12.6	100.0 ± 12.4	96.8 ± 12.5	96.9 ± 12.0	98.4 ± 12.7	0.019
Hip circumference (cm), mean ± SD	103.6 ± 11.3	100.3 ± 8.6	105.9 ± 12.3	102.5 ± 9.4	104.0 ± 11.7	0.007
Waist-hip ratio, mean ± SD	0.95 ± 0.08	1.00 ± 0.07	0.92 ± 0.07	0.94 ± 0.08	0.95 ± 0.08	0.437
Waist-to-height ratio, mean ± SD	0.63 ± 0.08	0.61 ± 0.07	0.65 ± 0.08	0.61 ± 0.07	0.64 ± 0.08	<0.001
Haemodynamic						
SBP (mmHg), mean ± SD	138.4 ± 20.9	140.6 ± 20.6	136.9 ± 21.0	135.8 ± 19.8	139.1 ± 21.2	0.004
DBP (mmHg), mean ± SD	78.9 ± 11.0	80.3 ± 11.5	78.0 ± 10.7	80.5 ± 11.1	78.5 ± 11.0	<0.001
Pulse pressure (mmHg), mean ± SD	59.5 ± 16.8	60.3 ± 16.4	58.9 ± 17.0	55.3 ± 14.1	60.6 ± 17.2	<0.001
MAP (mmHg), mean ± SD	98.7 ± 12.8	100.4 ± 13.0	97.6 ± 12.6	99.0 ± 13.0	98.7 ± 12.8	0.667
Blood biomarkers						
CRP (mg/L), median [IQR]	2.5 [1.2–5.0]	2.0 [1.0–4.1]	2.9 [1.4–5.7]	2.2 [1.0–3.9]	2.6 [1.3–5.4]	<0.001
HDL-C (mg/dL), mean ± SD	41.2 ± 10.4	39.1 ± 9.9	42.6 ± 10.4	41.4 ± 10.6	41.1 ± 10.3	0.649
Total cholesterol (mg/dL), mean ± SD	200.7 ± 46.4	197.7 ± 48.0	202.7 ± 45.2	204.1 ± 45.6	199.8 ± 46.6	0.091
TSH (μIU/mL), median [IQR]	1.90 [1.22–2.90]	1.89 [1.24–2.83]	1.90 [1.20–2.93]	1.88 [1.20–2.89]	1.91 [1.24–2.90]	0.596
25(OH)D (ng/mL), mean ± SD	24.4 ± 8.6	26.3 ± 9.2	23.0 ± 7.9	25.9 ± 9.8	23.9 ± 8.2	<0.001
Functional outcome						
Slow gait (<0.8 m/s), n (%)	1380 (70.4)	472 (60.1)	908 (77.4)	0 (0.0)	1380 (89.5)	—
Weak handgrip (EWGSOP2), n (%)	352 (18.0)	193 (24.5)	159 (13.6)	0 (0.0)	352 (22.8)	—
Impaired balance (<10 s one-leg stance), n (%)	684 (35.0)	222 (28.3)	462 (39.6)	0 (0.0)	684 (44.5)	—
≥1 functional deficit, n (%)	1543 (78.7)	555 (70.4)	988 (84.2)	0 (0.0)	1543 (100.0)	—
≥2 functional deficits, n (%)	702 (35.8)	250 (31.7)	452 (38.5)	0 (0.0)	702 (45.5)	—
All three deficits, n (%)	171 (8.7)	82 (10.4)	89 (7.6)	0 (0.0)	171 (11.1)	—

Values are mean ± SD, median [IQR], or n (%). *p*-values compare participants without and with ≥1 functional deficit (Welch *t*-test, Mann–Whitney U test, or χ^2^ test). FD was defined as ≥1 of slow gait (<0.8 m/s in the faster of two 2.5-m walks); weak handgrip (EWGSOP2 cut-points: <27 kg in men, <16 kg in women); impaired balance (inability to hold a one-leg stance for 10 s on either leg). Component percentages are computed among participants with that test observed. 25(OH)D = 25-hydroxyvitamin D; BMI = body mass index; CRP = C-reactive protein; DBP = diastolic blood pressure; HDL-C = high-density lipoprotein cholesterol; IQR = interquartile range; MAP = mean arterial pressure; SBP = systolic blood pressure; TSH = thyroid-stimulating hormone.

**Table 2:** Nested cross-validated performance of nine ML classifiers (N = 1961; 5 × 10 outer folds; ordered by AUC-ROC).

Classifier	Family	AUC-ROC (95% CI)	ΔAUC vs. LR (95% CI)	p_Holm_	Brier	Scaled Brier	Calib. Slope	AP (No Deficit)	Bal. Accuracy	Overfit Gap
Logistic Regression	Linear	0.751 (0.724–0.779)	Reference	—	0.147	0.125	0.96	0.441	0.681	0.011
Spline Logistic Regression	Linear	0.750 (0.721–0.778)	−0.002 (−0.010, +0.007)	1.000	0.147	0.126	1.03	0.441	0.681	0.021
Extra Trees	Ensemble	0.745 (0.714–0.776)	−0.006 (−0.022, +0.009)	1.000	0.148	0.119	1.22	0.434	0.687	0.149
Random Forest	Ensemble	0.744 (0.713–0.776)	−0.007 (−0.025, +0.011)	1.000	0.147	0.121	1.08	0.434	0.683	0.091
Gradient Boosting	Ensemble	0.739 (0.707–0.772)	−0.012 (−0.031, +0.007)	0.808	0.149	0.113	1.04	0.429	0.675	0.091
K-Nearest Neighbors	Instance	0.730 (0.698–0.762)	−0.021 (−0.039, −0.004)	0.089	0.150	0.105	1.19	0.419	0.666	0.270
Naïve Bayes	Probabilistic	0.723 (0.694–0.753)	−0.028 (−0.048, −0.009)	0.043	0.196	−0.166	0.31	0.417	0.656	0.010
AdaBoost	Ensemble	0.721 (0.690–0.752)	−0.030 (−0.053, −0.007)	0.065	0.157	0.065	2.10	0.377	0.638	0.027
Decision Tree	Tree	0.711 (0.679–0.743)	−0.041 (−0.068, −0.013)	0.043	0.152	0.092	0.79	0.355	0.652	0.032

Values are means across 50 outer folds. Ninety-five percent confidence intervals (CI) for AUC-ROC and paired differences versus Logistic Regression (ΔAUC) were obtained with the corrected resampled *t*-test; *p*-values are Holm-adjusted for eight comparisons. Scaled Brier = 1 − Brier/Brier_null_ (higher = better; Brier_null_ is the Brier score of a model assigning the training-fold prevalence to every participant). Calibration slope was estimated from pooled out-of-fold predictions (ideal = 1). AP (no deficit) = average precision for the no-deficit class (baseline = 0.213). Balanced accuracy (Bal. accuracy) at a threshold equal to the training-fold prevalence; Calib. slope = calibration slope. Overfit gap = training AUC − validation AUC. Hyperparameters were tuned within an inner stratified 5-fold loop minimizing log-loss. AP = average precision; AUC-ROC = area under the receiver-operating characteristic curve; LR = Logistic Regression; ML = machine learning.

## Data Availability

The data analyzed in this study originated from the 2012 Mexican Health and Aging Study (MHAS) biomarker sub-study and are publicly available from the MHAS website (www.MHASweb.org) upon registration. The analysis code is available from the corresponding author upon reasonable request.

## Abbreviations

The following abbreviations are used in this manuscript:

25(OH)D	25-hydroxyvitamin D
AP	Average precision
AUC	Area under the curve
AUC-ROC	Area under the receiver-operating characteristic curve
BMI	Body mass index
BP	Blood pressure
CRP	C-reactive protein
CV	Cross-validation
DBP	Diastolic blood pressure
DCA	Decision curve analysis
DT	Decision tree
ENSANUT	Encuesta Nacional de Salud y Nutrición
ET	Extra Trees
EWGSOP2	European Working Group on Sarcopenia in Older People 2
FD	Functional deficit
GB	Gradient boosting
HDL-C	High-density lipoprotein cholesterol
IQR	Interquartile range
K-NN	K-nearest neighbors
LR	Logistic regression
MAP	Mean arterial pressure
MCC	Matthews correlation coefficient
MDI	Mean decrease in impurity
MHAS	Mexican Health and Aging Study
ML	Machine learning
NB	Naïve Bayes
OOF	Out-of-fold
PP	Pulse pressure
PR	Precision–recall
RF	Random forest
ROC	Receiver-operating characteristic
SABE	Salud, Bienestar y Envejecimiento
SBP	Systolic blood pressure
SD	Standard deviation
SHAP	SHapley Additive exPlanations
Spline LR	Penalized spline logistic regression
TRIPOD	Transparent Reporting of a multivariable prediction model for Individual Prognosis Or Diagnosis
TRIPOD + AI	TRIPOD statement extension for prediction models using regression or machine learning
TSH	Thyroid-stimulating hormone
WHR	Waist-to-hip ratio
WtHR	Waist-to-height ratio
